# The determinants of crop productivity and its effect on food and nutrition security in rural communities of South Africa

**DOI:** 10.3389/fsufs.2023.1091333

**Published:** 2023-05-05

**Authors:** Simphiwe Innocentia Hlatshwayo, Mjabuliseni Simon Cloapas Ngidi, Temitope Oluwaseun Ojo, Albert Thembinkosi Modi, Tafadzwanashe Mabhaudhi, Rob Slotow

**Affiliations:** 1 African Centre for Food Security, School of Agricultural, Earth and Environmental Sciences, College of Agriculture, Engineering, and Science, University of KwaZulu-Natal, Pietermaritzburg, South Africa; 2 Centre for Transformative Agricultural and Food Systems, School of Agricultural, Earth and Environmental Sciences, College of Agriculture, Engineering, and Science, University of KwaZulu-Natal, Pietermaritzburg, South Africa; 3 Department of Agricultural Extension and Rural Resource Management, School of Agricultural, Earth and Environmental Sciences, College of Agriculture, Engineering, and Science, University of KwaZulu-Natal, Pietermaritzburg, South Africa; 4 Department of Agricultural Economics, Obafemi Awolowo University, Ile-Ife, Nigeria; 5 Disaster Management Training and Education Centre for Africa, University of the Free State, Bloemfontein, South Africa; 6 Centre for Transformative Agricultural and Food Systems, School of Life Sciences, College of Agriculture, Engineering, and Science, University of KwaZulu-Natal, Pietermaritzburg, South Africa

**Keywords:** smallholder farmers, crop productivity, food security, nutrition status, Household Food Insecurity Access Scale

## Abstract

**Introduction:**

High crop productivity has the potential to improve the food and nutrition security status of not only smallholder farmers but also households in general. However, smallholder farmers operate in a dynamic environment whereby their crop production is affected by various factors that hinder it from lessening food insecurity and malnutrition in rural areas. The study investigated the determinants of crop productivity and its effect on household food and nutrition security status in South Africa.

**Methods:**

This study employed a quantitative research method. A total of 1520 households were selected using the multi-stage stratified random sampling technique. Out of the total sample size of 1520, 386 were crop producers, 176 producers were from Mpumalanga province, and 210 producers were from the Limpopo province.

**Results and discussion:**

Most of the smallholder farmers do not have access to the irrigation system, mechanization, and agricultural inputs. The Household Food Insecurity Access Scale showed that most smallholder farmers were food insecure, with 78% of the farmers in each province found to be food insecure. The results from Household Dietary Diversity Score (HDDS) showed that in the overall sampled population, 50% of smallholder farmers had highly diverse diets. Only 50% of the smallholder farmers had high dietary diversity in each province. Irrigation systems and involvement in crop production had a positive influence on the crop productivity of smallholder farmers. The results from the Conditional Mixed Process (CMP) model showed that ownership of livestock, harvest, and disability in the family negatively impacted smallholder farmers’ food security status while household size had a positive effect on the food security of smallholder farmers. The results also showed that social grants, agricultural assistance, and harvest had a negative impact on the nutrition status of smallholder farmers. While household size had a positive impact on the nutrition status of smallholder farmers.

**Conclusion and recommendations:**

Factors such as irrigation systems and involvement in crop production influenced crop productivity. Household size influenced the nutritional status of smallholder farmers while harvest size affected the food security status. There is a need to encourage more households to get involved in farming. Government and nongovernmental organizations need to support smallholder farmers with agricultural productive resources like irrigation systems to improve their crop productivity.

## 1 Introduction

The agricultural industry is the most critical contributor to African developing countries’ economies and rural livelihoods (Mwadalu and Mwangi, 2013). It generates 35% of the continent’s Gross Domestic Product (GDP), 40% of export revenues, and 70% of employment (Nyage et al., 2011). Furthermore, over 75% of the food consumed in the country is produced by small-scale farmers [Food and Agriculture Organisation (FAO), 2015]. An estimated 70% of South Africans who live in rural areas depend on agriculture and are engaged in smallholder subsistence farming. This emphasizes the significance of smallholder farmers, as well as agriculture in general, in South Africa. Despite the fact that agriculture is the most important sector of the South African economy in terms of contribution to livelihoods, job creation, GDP, and export profits, the country continues to face malnutrition, hunger, and food insecurity, particularly in rural regions. At the national level, South Africa is regarded as food secure, whereas a significant proportion (30–60%) of rural households is food insecure [Statistics South Africa (STATS SA), 2018]. In 2018, ~ 11.3% of families and 9.7% of people were food insecure [Statistics South Africa (STATS SA), 2018]. Approximately 68% of South African women and 31% of South African men were obese or overweight in 2022 (Western Cape Government, 2022). Furthermore, United Nations South Africa (2021) reported that ~ 27% of children younger than 5 years were stunted.

South African smallholder farmers are still stuck in low-productivity traditional technologies that have a negative impact on livelihoods and output (Obi and Seleka, 2011; Calzadilla et al., 2014). Low agricultural productivity threatens the efforts of lessening poverty and improving food security. It also limits the ability of farmers to take on new opportunities that exist in the worldwide food system (Turpie and Visser, 2013). In 2050, it is estimated that the global population will increase to 9 billion, and food demand is expected to rise by 60% (Goldblatt, 2010). To provide enough food for this rapidly cumulative population under existing changes in climate and social and land use, sustainable agricultural productivity should be utilized in food production (Goldblatt, 2010). Increased food demand can be satisfied by increased production or agricultural expansion. Crop productivity can be increased by employing fertilizers, innovative irrigation, and the use of sustainable farming methods (Elferink and Schierhorn, 2016). This can help to boost crop yields from existing land by making better use of available resources and inputs.

The agricultural sector plays a critical role in increasing crop productivity and achieving food security (Otsuka, 2013; Wegren and Elvestad, 2018). However, smallholder farmers are unable to fully adopt the existing technology and knowledge due to various challenges. There are sustainable agricultural practices that smallholder farmers can adopt in order to improve crop productivity; the practices include crop cover, intercropping, minimum tillage, crop rotation, green and animal manure, crop rotation, and rainwater harvesting (Myeni et al., 2019). However, the intensity of adoption of these practices by South African smallholder farmers is still minimal. Farmers continue to produce small plots despite possessing small land; hence, the land stays unused (1–2 hectares). Previous studies conducted in South Africa and across the African continent found that socioeconomic factors, farm features, and agro-climatic zone characteristics were the most critical determinants impacting crop productivity among smallholder farmers (Obasi et al., 2013; Mango et al., 2017; Mekuriaw et al., 2018;Myeni et al., 2019). These characteristics include cropping patterns, years of farming experience, age, a lack of financing, the use of low-input technologies, a lack of knowledge of high-input technologies, inadequate farm management skills, as well as poor extension services, and high cost. Therefore, factors influencing crop productivity differ with provinces and areas as a result of differences in natural resources, access to education, cultural beliefs, and sufficient information on sustainable farming methods, infrastructure, and extension services (Pretty et al., 2011; Mungai et al., 2016).

Understanding the role of crop productivity on food security across South Africa is critical to develop interventions to improve crop productivity and adopt appropriate agricultural methods, considering the vast diversity of South African regions. Despite the crucial role of agricultural crop productivity in improving food security in many developing countries such as South Africa, empirical research on the links between the two is scarce. Several studies have paid more attention to analyzing factors that determine crop productivity among smallholder farmers in several developing countries (Obasi et al., 2013; Mango et al.,2017; Mekuriaw et al., 2018; Myeni et al., 2019). On one hand, the food security studies (Sinyolo et al., 2014; Musemwa et al.,2015; Walsh and Van Rooyen, 2015; Sinyolo and Mudhara, 2018) have not investigated the role of crop productivity. As a result, quantitative research relating crop productivity and poverty indicators such as food insecurity is required in order to provide empirical proof of the role agricultural crop productivity plays in rural food insecurity reduction. South African agriculture is dual with commercial farmers dominating the sector (Amelework et al., 2021). Government and policymakers are engaged in finding pathways to improve the productivity of smallholder farmers to enhance their contribution to the economy and food security.

Understanding factors influencing crop productivity is essential for policymakers in the country. It is against this backdrop that the study seeks to explore the factors influencing crop productivity and its effect on household food and nutrition security in South Africa. The study intends to reduce the existing gap by evaluating the relationship between crop production competence and food and nutrition security status among smallholder farming households in Limpopo and Mpumalanga, South Africa.

## 2 Research methodology

### 2.1 Description of study areas

The study used secondary data which were obtained from the nine different provinces of South Africa. However, this study focused on only two provinces (Mpumalanga and Limpopo). These provinces were selected in this study, as they are listed as the top three poorest provinces in South Africa, together with the Eastern Cape [Statistics South Africa (STATS SA), 2019]. Unemployment, poverty, and income inequality in these provinces differentiate them from other provinces [Statistics South Africa (STATS SA), 2019]. All these conditions are prominent in rural households. Empirical evidence revealed that the majority of rural households rely on agriculture activities for livelihood and food security purposes.

Limpopo province has a sub-tropical climate which mostly occurs in a summer-rainfall season (October to March). The province receives rainfall that ranges between 300 and 600 mm annually. The average temperature ranges between°C and 20°C in winter and 7 and 27°C in summer (Mzezewa et al., 2010). During the winter season, mornings are very cold and dry with sunny days, and nights are typically frost-free and cold (Tshiala et al., 2011). The province is a major agricultural region in the country, producing vegetables, tea, cereals, and fruits.

Mpumalanga province has two main regions, the sub-tropical Lowveld plains and Highveld escarpment. The Highveld is much cooler, as it receives an altitude of 1,700–2,300m above the sea level, while the Lowveld is subtropical because of its closeness to the hot Indian Ocean. The Lowveld is relatively hot in summer and warm in winter while the Highveld is warm in summer but cold in winter (Cadman, 2007). The province receives temperature that ranges from 6 to 20°C in winter and 20 to 38°C in summer (Benhin, 2006). The province encompasses the production of sugar cane and crops such as maize, wheat, potatoes, nuts, sunflower, sub-tropical and tropical fruits, and livestock production (NAFCOC, 2014).

### 2.2 Data collection method

The secondary data used in this study were collected in the season of 2016/2017, with the aim of understanding smallholder farmers’ crop production, market access opportunities, and food and nutrition security status in different parts of rural areas in South Africa. Smallholder farmers were interviewed using a questionnaire that consist of both close-ended and open-ended questions. The quantitative research method was used to choose a total of 1,520 smallholder farmers using the multi-stage stratified random sampling technique. According to Mujere (2016), in stratified random sampling, the population is made up of nonoverlapping sub-populations (named strata) that differ from one another in terms of selected variables. Stratified random sampling ensures that important strata are well represented using a smaller sample that is less costly and requires less time (Mujere, 2016). In this study, Mpumalanga was divided into four districts, and Limpopo was divided into three districts. The variation in different groups was based on socioeconomic variables, such as gender and age. The data were collected by the Department of Agriculture, Land Reform, and Rural Development (DALRRD) under the South African Vulnerability Assessment Committee (SAVAC).

### 2.3 Methods of data analysis

The study aimed to quantify the impact that crop productivity has on the food and nutrition security of smallholder farmers. In this study, crop productivity is defined as the ability of farmers to efficiently allocate the inputs they have to produce economic outputs. Food and nutrition security is referred to as the state of having constant physical, social, and economic access to food in adequate quantity and quality to suit one’s dietary needs and food preferences. It is assumed that farmers with high crop productivity are food and nutrition secure. The household food insecurity access scale (HFIAS) and the household food dietary diversity score (HDDS) were used in the food security assessment. The HDDS includes the range of food and dietary diversity available to a household. Dietary variety statistics are gathered by asking 24-h recall questions on the food groups consumed by a household. Swindale and Bilinsky (2006) established the conventional 12 food groups. Cereal, fish and seafood, meat, roots and tubers, poultry and eggs, vegetables, fruits, sugar/honey, oil/fats, pulses/legumes/nuts, milk and milk products, and miscellaneous (which includes spices, sauces, salt, and other condiments) are the 12 food groups. The HDDS in this study was used as an outcome/dependent variable to show nutrition diversity among crop producers and non-crop producers.

The food access dimension of food insecurity was evaluated usingthe Household Food Insecurity Access Scale (HFIAS) (Coates et al., 2007). The HFIAS entails nine questions based on an experience of food insecurity happening within the past 4 weeks. The questions are grouped into three domains that illustrate the core experiences as follows: uncertainty about food supply, lack of food quality, and inadequate food intake (Coates et al., 2007). The tool has a standard scoring procedure, where 1 represents occurrence and 0 represents non-occurrence. The frequency scores in this study ranged from 0 to 3, with 0 indicating non-occurrence, 1 indicating rarely (one or two times in the last month), 2 indicating sometimes (three to ten times in the past month), and 3 indicating regularly (more than ten times in 30 days).

The study employed the Tobit regression model to quantify the magnitude and direction of the effects of the factors influencing the crop productivity of smallholder farmers. The model was previously used by many studies (Ele et al., 2013; Rubhara and Mudhara, 2019; Rubhara et al., 2020). The model addresses the factors influencing the decision to participate in crop production and the extent of crop production, assuming that both decisions are influenced by the same set of variables (Buke, 2009). The Tobit model was suitable for analyzing variables with lower and upper bounds (McDonald and Moffitt, 1980). The dependent variable, crop productivity is upper censored at 1 and lower censored at 0 in this case since it can take values ranging between 0 and 1. Rural households that do not grow crops have a crop productivity of 0; farmers who do grow crops have a crop productivity of 1. The model was used to estimate linear relationships since the dependent variable is censored from the left to the right (Tobin, 1958). Unlike the ordinary least squares regression method, the Tobit model matches the data well since it considers the qualitative difference between zero and continuous values (Bukenya, 2017; Oduniyi, 2018).

The Tobit model is defined as follows:

(1) $$Y_{i}^{*}=\beta_{0}+\beta X_{i}+\varepsilon_{i}$$

(2) $$\begin{array}{l} Y_{i}^{*}=1\;if\;Y_{i}^{*}>0\\ Y_{i}^{*}=0\;if\;Y_{i}^{*}\leq 0 \end{array}$$

(3) $$Y_{i}^{*}=0\;if\;Y_{i}=0$$

where $Y_{i}^{*}$ is the dependent variable’s latent variable, *β* is the vector of parameters to be assessed, *X_i_* is a collection of explanatory variables, and *ε_i_* is the disturbance. The errors in the model are considered to be independent, *N* (0, *σ*^2^)distributed, and conditional on *X_i_*. The observe $Y_{i}^{*}$ is denoted as 1 $Y_{i}^{*}>0$, as well as 0 $Y_{i}^{*}\leq 0$.

To investigate the impact of crop productivity on the nutrition and food security of smallholder farmers in various segments of South Africa’s Limpopo and Mpumalanga provinces, we estimated the following equation, using the subsistence farmer as the unit of analysis:

(4) $$y_{i}=\beta_{0}+\beta_{1}\times CP_{i}+\beta_{2}\chi_{i}+\eta_{i}+v_{i}$$

where *y_i_* is an indicator of a smallholder farmer’s *i* food security or nutritional status; *CP_i_* is a binary variable that takes 1 if the smallholder farmer *i* had crop productivity and 0 otherwise; *χ_i_* is a vector of household or farm level characteristics; *η_i_* is a term that describes unobserved heterogeneity which is expected to be unrelated to the explanatory variables vector *χ_i_* and relates to every smallholder farmer residing at the same location, and *v_i_* conveys the rest of variation with *v_i_* ~ *IIDN* (0,1).

If the vector *χ_i_* includes all of the elements hypothesized to influence crop productivity, including location-fixed effects, and is uncorrelated with the error*v_i_*, therefore an ordinary least squares (OLS) regression of Eq. (4) will produce accurate results. In such a scenario, the coefficient of interest *β*_1_, which measures the effect of crop productivity extent, can be viewed as the actual impact of crop productivity on the nutritional and food security status of smallholder farmers.

The crop productivity of rural smallholder farmers is influenced by several unobserved variables, making it an endogenous variable, and the inability to account for this endogeneity might lead to biased and inconsistent findings. Crop productivity endogeneity bias occurs when some households possess resources and skills to improve crop productivity, whereas others do not. In a regression model shown in Eq. (4), the type of selection bias can overstate the real effect on crop productivity. On the contrary, poor and deprived smallholder farmers may be unable to increase crop productivity due to inadequate agricultural production resources. In this instance, failure to incorporate this type of bias will minimize the actual advantage of crop productivity. Crop productivity (CP), a possibly endogenous factor, is expressed as follows:

(5) $$CP_{i}^{*}=\alpha_{0}+\alpha_{1}Z_{i}+\alpha_{2}\chi_{i}+\eta_{2}+\varepsilon_{i}$$

where $CP_{i}^{*}$ is the propensity to increase crop productivity. However, $CP_{i}^{*}$ is unobserved and what we observe instead is the following:

(6) CP={1if Crop productivity score>00otherwise

The vector *ζ_i_* comprises a set of variables that influence crop productivity such as management and technical abilities of smallholder farmers and agricultural assistance from the government (Abdulai and Huffman, 2014; Manda et al., 2016). *η*_2_ is the unobserved heterogeneity component, which is not correlated with the vector of explanatory factors (*χ_i_*), and *ε_i_* represents the underlying unobserved variability. The unobserved heterogeneity components’ subscripts {1, 2} are equation indicators.

In the research literature, the conventional method for controlling endogeneity bias is to estimate Eq. (4) with crop productivity instrumental variables [Eq. (5)]. Instrumental variables are those that are substantially linked with the endogenous variable (crop productivity in this case) but not with unobserved factors that may influence the outcome variables (Angrist and Krueger, 2001). However, as is widely known, obtaining a good instrument is quite challenging. We estimate Eqs. (4) and (5) jointly to avoid issues that are closely correlated with imperfect instruments.

As previously noted, crop productivity’s endogeneity can considerably overestimate or underestimate the effect of crop productivity on nutrition and food security status. To account for this likelihood, we estimate Eqs. (4) and (5) jointly within Roodman (2011) Conditional (recursive) Mixed Process (CMP) framework. Other previous studies have used the CMP (Makate et al., 2016; Alhassan et al., 2020; Melesse et al., 2021). By allowing for a crossequation connection of the error terms and begin with a seemingly unrelated regression framework, the CMP accounts for selection bias caused by unobserved factors that affect our outcome variables. Allowing for crop productivity endogeneity in Eq. (7), we may express the joint, marginal likelihood as follows:

(7) $$\underset{\eta_{2}}{\int }\underset{\eta_{1}}{\int }\left[\prod^{\text{​}}L_{2}(\eta_{2})\prod^{\text{​}}L_{1}(\eta_{1})\right]f(\eta_{2},\eta_{1})d\eta_{2}\eta_{1}$$

where *L*_1_ and *L*_2_ are the conditional likelihood functions of Eqs. (4) and (5), respectively; *f* (*η*_2_, *η*_1_) is the joint distribution of the unseen heterogeneity factors. In this instance, the joint distribution of the unobserved effectsf (*η*_2_, *η*_1_) is assumed to be a two-dimensional normal distribution characterized as follows:

(8) $$\left(\begin{array}{l} \eta_{2}\\ \eta_{1} \end{array}\right)∼N\left(\left[\begin{array}{l} 0\\ 0 \end{array}\right],\left[\begin{array}{l} \sigma_{2}^{2}\\ \rho_{12}\sigma_{2}\sigma_{1}\sigma_{1}^{2} \end{array}\right]\right)$$

The conditional mixed process (CMP), which employs the Geweke, Hajivassiliou, and Keane (GHK) algorithm to reliably estimate the likelihood function specified in the specification or full model, is used to jointly estimate the complete specification or full model (Eq. 6). The fundamental purpose for evaluating Eqs. (3) and (4) jointly is to account for any self-selection bias. According to Maitra (2004), joint estimation indicates the probability of non-zero covariance between the error terms of Eqs. (3) and (4), i.e.,cov (*η*_2_, *η*_1_) ≠ 0. However, as we condition on the heterogeneity factors, Eqs. (3) and (4) become independent, making the probability function (6) above simply derived by basically multiplying the specific conditional likelihood functions of Eqs. (3) and (4) (Chamberlain and Griliches, 1975). Due to the difficulties in identifying acceptable instrumental variables, the joint model (with correlated errors) enables us to produce selection bias-corrected estimates for smallholder crop productivity and food and nutrition security status.

### 2.4 Variable description and statistics

The quantitative data were analyzed using STATA software (version 13). The descriptive statistics were performed to provide the key socioeconomic characteristics of the sampled smallholders. It was performed to show mean averages and percentages of the different factors that affect the crop productivity of smallholder farmers. The crop productivity of smallholder farmers is influenced by factors that can be grouped as internal, external, and sociodemographic factors. Sociodemographic factors include variables such as age of the household head, gender, household size, marital status, and educational level. Internal factors or household assets include off-farm income, number of cattle, irrigation system, and land. The level of access to information is taken by access to extension services which is the external factor. Table 1 shows a summary of the explanatory variables which were likely to influence the crop productivity of smallholder farmers.

## 3 Results

### 3.1 Descriptive results

#### 3.1.1 Demographic characteristics of smallholder farmers in relation to crop productivity

Smallholder farmers are characterized by many different sociodemographic factors, as shown in Tables 2, 3. The results showed that out of 609 smallholder farmers in Mpumalanga, 176 were crop producers and 433 were non-crop producers. The results also showed that out of 911 smallholder farmers in Limpopo province, 210 were crop producers and 701 were non-crop producers (Table 2).

Table 3 presents the demographic characteristic variations among crop and non-crop producers. The results showed that 74% of the crop producers knew about crop rotation while 26% did not know about it. Among non-crop producers, 88% knew about crop rotation while 12% did not know. In terms of access to irrigation, 48% crop producers had access while 52% did not have access. Among non-crop producers, only 6% had access to irrigation while 94% did not have access. Regarding access to agricultural inputs, 65% of crop producers had access while 35% did not have access. Among non-crop producers, only 18% had access to agricultural inputs while 82% did not have access. The results also showed that 22% of crop producers had access to mechanization while 78% did not have access. Among non-crop producers, 12% had access to mechanization while 88% did not have access.

#### 3.1.2 Prevalence of food insecurity by household characteristics based on HFIAS categories

**Figure 1:**
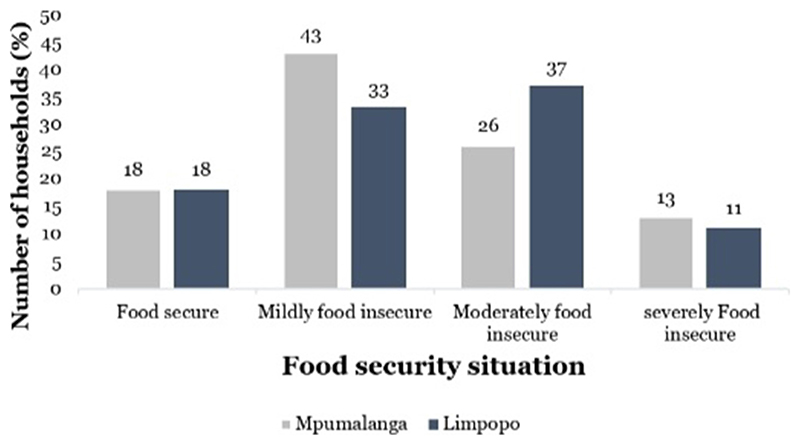
The food insecurity situation of the smallholder farmers in the two provinces [Mpumalanga (*n* = 609) and Limpopo (*n* = 911)]. Source: own analysis.

Figure 1 presents the fraction of the occurrence of food insecurity among the sampled smallholder farmers in the two provinces (Mpumalanga and Limpopo). The results revealed that in Mpumalanga province, 18% of the smallholder farmers were food secure. In the same province, ~ 43% of the smallholder farmers were mildly food insecure, followed by 26% of farmers who were moderately food insecure while 13% were found to be severely food insecure. In Limpopo province, 18% of smallholder farmers were food secure, 37% of smallholder farmers were moderately food insecure, 33% of the farmers were mildly food insecure, and 11% of the farmers were severely food insecure. The findings demonstrate that smallholder farmers in the two provinces experienced difficulties in accessing food, with 78% of the smallholder farmers from each found to be food insecure.

#### 3.1.3 Dietary diversity of smallholder farmers

**Figure 2:**
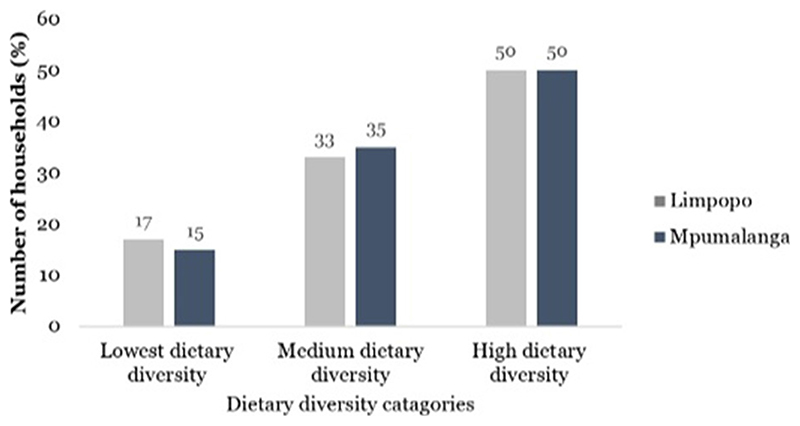
Dietary diversity of smallholder farmers in Limpopo (*n* = 911) and Mpumalanga (*n*= 609) provinces, South Africa.

Figure 2 shows the dietary diversity of smallholder farmers in the Mpumalanga and Limpopo provinces. Using the cutoffs recommended by Kennedy et al. (2011), ~ 50% of the smallholder farmers in the total sample had highly diverse diets, suggesting that six or more food groups were consumed by these smallholder farmers. Approximately 25% of the smallholder farmers had medium dietary diversity, and only 18% had low-diverse diets with less than three food groups consumed. In the provinces, both Mpumalanga and Limpopo each had 50% of smallholder farmers who had high-diverse diets. Approximately 35% and 33% of smallholder farmers consumed medium dietary diversity (4–5 food groups) in Mpumalanga and Limpopo provinces, respectively. Mpumalanga province had 15% of smallholder farmers with low diverse diets (less or equal to three food groups) while Mpumalanga had 17%.

### 3.2 Determinants of crop productivity under smallholder farming

Table 4 shows the results of the factors influencing the crop productivity of smallholder farmers. Irrigation system, involvement in crop production, and wealth index all positively influenced crop productivity (p<0.01). The results showed that access to agricultural assistance had a negative and significant impact on the crop productivity of smallholder farmers.

### 3.3 Effect of crop productivity on the HDDS (nutritional status) and HFAIS (food security) of smallholder farmers—Conditional mixed process model

Table 5 shows the estimation results from combining Eqs. (3) and (4). As previously stated, joint estimation of the system of equations enables us to account for endogeneity bias caused by crop productivity selectivity bias in the equations nutrition status (HDDS) and food security (HFIAS). The atanhrho shown at the end of Table 5 is a measure of selection bias. The identified atanhrho value is the arc-hyperbolic tangent of the rhos (p) to make them unbounded. A positive atanhrho value suggests that there are some unseen factors influencing crop productivity and the main outcome variable. The atanhrho_ 12 was significant (p<0.10) and negative in the outcome equations in this study. The negative result of atanhrho_ 12 indicates that no omitted variables influenced both outcome variables.

The CMP model results in Table 5 revealed that household size positively impacted both nutrition status (HDDS) and food security (HFAIS). The findings revealed that social grants and agricultural support had a negative and substantial effect on smallholder farmers’ nutrition status (HDDS). Smallholder farmers’ food security was negatively impacted by livestock ownership. The study obtained surprising results about the impact of harvest. Smallholder farmers’ overall harvest had a negative and significant impact on their nutrition status and food security. Disability in the family had a negative and significant (P <0.05) impact on smallholder farmers’ food security.

## 4 Discussion

The involvement of smallholder farmers in crop production helps to reduce poverty, food insecurity, and unemployment. The result of this study has proven that the involvement of crop production by smallholder farmers had a positive impact on crop productivity. This is because smallholder farmers are involved in crop production, and they are able to produce more for consumption and sell more to generate income. They are able to use all the opportunities they have to increase their productivity. This result was supported by Mathenge et al. (2010) and Mulenga et al. (2021), who reported that smallholder farmers who are involved in crop production can escape most of the unfavorable conditions they are faced with such as hunger, malnutrition, and unemployment. On the contrary, the wealth index of smallholder farmers showed a negative and significant influence. This happens because some farmers are unaware of their resources and living standards, causing them to under-utilize what they possess and produce inefficiently.

The irrigation system is an essential production input in agriculture. It allows farmers to have water for their crops in a desired manner and helps avoid dependency on rainfall. The result of this study has proven that irrigation system positively influenced crop productivity. This is because farmers can have access to stored water at any time they want and are able to grow any crop. This result was similar to that of Oni et al. (2011) and Sinyolo et al.(2014) who reported that irrigation systems had a positive role in rural households’ welfare and food security. However, most of the studies reported that the majority of smallholder farmers do not have access to an irrigation system and those who have them perform poorly (Denison and Manona, 2007; Fanadzo et al., 2010; Post et al., 2012). These studies reported that most smallholder farmers do not have adequate cropping systems, management practices, and irrigation application which led them using any type of irrigation without looking at the crop type and growth stage.

The positive impact of household size on nutrition status and food security is attributed to the fact that smallholder farming mainly depends on family labor, so an increase in household size leads to increased crop productivity as work is shared on the farm. On the contrary, Olayemi (2012) and Aidoo et al. (2013) found that household size had a negative and significant impact on food security.Olayemi (2012) reported that a large family size also means more children, which affects the dependency ratio and quantity of food intake. Therefore, the authors concluded that the larger the household size, the lesser the availability of food for each person within the family, and the nutritional status would be negatively affected. Aidoo et al. (2013) stated that an increase in household size means more family members to feed and indirectly decreases income per household head and increases expenditure per head and food consumption.

Social grants showed a negative impact on the HDDS, implying that the probability of nutritional status decreases with an increase in social grants among smallholder farmers. The possible explanation is that some smallholder farmers who receive social grants do not want to be involved in crop production and do not purchase healthy food with their funds. In line with this result, Boone et al. (2013) and Sinyolo et al. (2017) explained that social grant is a disincentive for many households to participate in farming activities. Most rural households consider social grants as their major source of income and ignore farming. This led to less dietary diversity in the food they consume since the social grant is not enough to improve their nutrition. However, Waidler and Devereux (2019) found that the old-age pension grant had a positive and significant impact on the dietary diversity index of households. The study explained that the old age pension grant is higher than the child support grant and is expected to benefit the whole household and have a high impact on household food security.

Ownership of livestock in smallholder farming has the potential to improve food security by raising the incomes of the unprivileged and by increasing the accessibility of nutrient-dense foods (Jodlowski et al., 2016). However, in this study, ownership of livestock had a negative impact on food security. This is because in most cases, farmers with livestock do not want to sell their livestock, they want to keep it for a long time, and this leaves them susceptible to food insecurity. This result was in line with Kyaw et al. (2018), who reported that smallholder farmers usually have inadequate land for both crop and livestock production, so they end up compromising crop production. This led to less crop production which result in hunger, malnutrition, and food insecurity. On the contrary, Jodlowski et al. (2016) found that ownership of livestock improved the dietary diversity of households in Zambia through direct consumption of animal products produced on the farm and through increased consumption expenditures.

The agricultural extension services are intended to boost agricultural productivity among smallholder farmers, which, in turn, increases their food security and improves rural livelihoods. However, in this study, the result showed that agricultural assistance from the government had a negative impact on the nutrition status (HDDS) and crop productivity of smallholder farmers. This is because most of the smallholder farmers reside in remote areas where development is very slow, and they end up not benefiting from some of the extension programs. However, in Malawi, households that found extension services very useful were associated with high farm productivity and food security (Ragasa and Mazunda, 2018). Furthermore, Pan et al. (2018) found that in Uganda, farmers who were involved in agricultural extension programs could use better cultivation methods and achieve improved food security. Nevertheless, Aliber and Hall (2012) and Kruger and Gilles (2014) confirmed the observed result; they reported that there is poor performance of extension service in many rural areas. The authors further explained that this is due to many factors including administrative inefficiency, deficient program design, and weaknesses in information delivery systems.

Harvest in this study referred to the output/yield that smallholder farmers get during their crop production. It is the amount of harvest that determines if farmers will be able to consume and sell. In this study, the result showed that harvest had a negative effect on both the nutrition status and food security of smallholder farmers. This is because most of the smallholder farmers did not produce enough for consumption and to sell. This caused them to only consume what they have and not be able to get income to buy other nutritious food groups. On the contrary, Liliane and Charles (2020) found that crop yields significantly reduced poverty and malnutrition. The study further explained that crop yield is influenced by numerous factors in a specific area. The factors include managerial decisions, climatic conditions, agricultural practices, diseases and pests, soil fertility, and topography. So, it is crucial to ensure that farmers understand all these factors before they commence crop production.

Having a disabled member in the family or living with disabilities has a profound effect on food and nutrition security, especially in the parts of the world where the majority of the people rely on agriculture for a living. Wolbring and Mackay (2014) reported that disabled people are part of the public, and they are the group that mostly experiences high levels of food insecurity. This was confirmed in this study as the study found that having a disabled family member had a negative impact on the food security of smallholder farmers. This implies that having a disabled family member increases stress and affects the mental and physical health of other family members. This affects their decision to be involved in crop production and that affects their food security. Gomda (2018) identified two types of participation of people with disabilities in agriculture ‘participation through labor contribution’ and participation through decision making’. The author also found that people with disabilities were associated with a high level of food insecurity. This was because the extension services delivery was not addressing the peculiar needs of disabled farmers.

## 5 Conclusion and recommendations

The primary objective of this study was to determine the impact of crop productivity on household food and nutrition security status in South Africa. The descriptive results showed that most of the smallholder farmers have inadequate access to irrigation system, mechanization, and agricultural inputs. The results from the CMP model showed that ownership of livestock, harvest, and disability in the family had a negative impact on the food security of smallholder farmers. While the household size and family member with HIV had a positive impact on the food security of smallholder farmers. The results also showed that social grant, agricultural assistance, and harvest had a negative impact on the nutrition status of smallholder farmers, while household size had a positive impact on the nutrition status of smallholder farmers.

The results that were received in the study showed that determinants of crop productivity had an impact on the food security and nutrition status of smallholder farmers. An improvement in these determinants can lead to an improvement in food security and nutrition status. To increase the harvest for smallholders, farmers need to be trained on how to efficiently manage the resources that they have. They need to be trained on all the factors (farming methods, fertilizer application) that affect their yield so that they can produce more. This can be done by hiring extension workers. The government needs to hire enough skilled extension workers who are capable of training farmers. Extension workers need to do more workshops and training in rural areas to increase awareness of food security and nutrition status. This will help farmers to change their style of living and improve their crop productivity. The workshops will also help farmers on how to use the social grant as one of the incentives to be involved in crop production. They need to be taught on how their social grant can help them to acquire inputs that are needed for farming and what benefits they will receive from doing that. In addition, the workshops can be used to train and teach smallholder farmers how to cope mentally and physically when they stay with a disabled person. Farmers can also be taught on how to allocate their time effectively.

## Figures and Tables

**Table 1:** Factors that are estimated to affect crop productivity.

Variable name	Variable definition	Variable type and measurement
Age	Age of the household head	In years (continuous)
Gender	Gender of household head	If the participant is male, 1 is assigned; otherwise, 0 is assigned.
Marriage	Marriage status of the household head	1 = if the respondent is married, 0 otherwise
Household size	Number of family members	Size of household (continuous)
Level of education	Education level of the household head	Years of education (continuous)
Yield	Yield harvested	In kilometers (continuous)
Land	Size of land cultivated	In Ha (continuous)
Involvement in crop production	Household head involved in crop production	1 = if respondents had involved in crop production, 0 otherwise
Irrigation	Access to irrigation scheme	If the participant had exposure to an irrigation system, 1 was assigned; otherwise, 0 was assigned.
Family member with HIV	Family member that is living with HIV	If there is an HIV-positive member of the family, 1; otherwise, 0.
Family member worked on farm	Family member worked on farm	If there is a member who worked on the farm, 1; otherwise, 0.
Disability in the family	Family member with disability	If there is a disabled member of the family, 1 is assigned; otherwise, 0 is assigned.
Livestock	Ownership of livestock	If the responder had livestock, 1; otherwise, 0.
Social grant	Social grant received by households from government	If a family member obtained a social grant, 1 is assigned; otherwise, 0 is assigned.
Agricultural assistance	Access to extension service	If participants have access to extension services, they received a 1; otherwise, they received a 0.

Source: own analysis.

**Table 2:** Demographic features of smallholder farmers in South Africa’s Limpopo and Mpumalanga provinces.

		Crop producers	Non-crop producers	Total
Province name	Mpumalanga	176	433	609
	Limpopo	210	701	911
Total		386	1,134	1,520

Source: own analysis.

**Table 3:** Demographic features of smallholder farmers in South Africa’s Limpopo and Mpumalanga provinces.

Variables	Crop producers (386)		Non-crop producers (1,134)		Overall Freq
	%	Freq	%	Freq	
Crop rotation					
Yes	74	286	88	1,000	1,286
No	26	100	12	134	234
Access to irrigation					
Yes	48	186	6	66	252
No	52	200	94	1,068	1,268
Access to inputs					
Yes	65	250	18	200	450
No	35	136	82	934	1,070
Access to mechanization					
Yes	22	86	12	134	220
No	78	300	88	1,000	1,300

Source: own analysis.

**Table 4:** Determinants of crop productivity under smallholder farming.

Variables	Coef.	Std.Err.	*p*-Value
Age of the household head	2.867	14.700	0.845
Household size	–79.950	91.045	0.380
Gender of the household head	3,712.648	1,101.649	0.678
Educational level of household head	907.609	3,353.573	0.787
Marital status	–3,972.112	4,957.777	0.423
Crop number	–87.154	193.286	0.652
Irrigation system	11,487.926	1,967.006	0.000***
Access to agricultural assistance	–3,417.430	807.491	0.000***
Involvement in crop production	9,047.278	3,212.759	0.005***
Family member worked on farm	–1,382.487	2,250.941	0.539
Social grant	–1,723.233	1,296.330	0.184
WEATHINDEX	–3,875.732	1,301.283	0.003***
Access to extensional advises	609.984	908.477	0.502
Disability in the family	7,078.643	5,462.066	0.195
Family member with HIV	4,225.499	2,431.313	0.663
Constant	–22,800.000	4,630.378	0.000***
var (e. harvest in kg)	70,200,000.000	3,390,000.000	.b
Mean dependent var	383.280	SD dependent var	6,715.550
Pseudo r-squared	0.004	Number of obs	1,424.000
Chi-square	73.845	Prob > chi2	0.000
Akaike crit. (AIC)	18,863.616	Bayesian crit. (BIC)	18,958.318

*** Indicate significance at 1% level. Source: Authors’ own analysis.

**Table 5:** Determinants of crop productivity on nutritional status and food security using the Conditional Mixed Process (CMP) model.

Variables	HHDS (nutritional status)			HFAIS (food security)		
	Coef.	Std.Err.	P-value	Coef.	Std.Err.	P-value
Age	–0.002	0.002	0.412	–0.003	0.002	0.161
Household size	0.052	0.016	0.001***	0.071	0.013	0.000***
Social grant	–0.254	0.144	0.077*	–0.667	0.431	0.122
Agricultural assistance	–0.647	0.167	0.000***	–0.103	0.119	0.388
Ownership of livestock	0.187	0.476	0.694	–0.795	0.354	0.025**
Educational level of household head	0.176	0.590	0.765	0.632	0.487	0.194
Government advice	–0.203	0.175	0.246	0.151	0.132	0.250
Harvest (kg)	–0.030	0.016	0.051**	–0.027	0.014	0.050**
Disability in the family	0.300	0.467	0.520	–1.658	0.785	0.035**
Economic activity	0.355	0.219	0.106	1.510	0.760	0.147
Gender	0.849	0.682	0.213	0.378	0.192	0.213
Family member with HIV	0.029	0.407	0.943	0.645	0.302	0.465
/cut_2_1	–0.859	0.167	0.000			
/cut_2_2	1.663	0.172	0.000			
/atanhrho_12	–0.081	0.048	0.090			
rho_12	–0.080	–0.172	0.013		

***, **, *Indicate significance at 1, 5, and 10% level, respectively.

## Data Availability

The data analyzed in this study is subject to the following licenses/restrictions: Restriction apply to the availability of these data. Data was obtained from the Department of Agriculture, Land Reform, and Rural Development (DALRRD) and are available from South African Vulnerability Assessment Committee (SAVAC) secretariat with the permission of Department of Agriculture, Land Reform, and Rural Development (DALRRD). Requests to access these datasets should be directed to www.dalrrd.gov.za.
